# Sex and Heart Failure Treatment Prescription and Adherence

**DOI:** 10.3389/fcvm.2021.630141

**Published:** 2021-05-07

**Authors:** Marta Farrero, Lavanya Bellumkonda, Inés Gómez Otero, Beatriz Díaz Molina

**Affiliations:** ^1^Heart Failure Unit, Cardiology, Hospital Clínic Barcelona, Barcelona, Spain; ^2^Section of Cardiovascular Medicine, Department of Internal Medicine, Yale University School of Medicine, New Haven, CT, United States; ^3^Heart Failure Unit, Cardiology, University Clinical Hospital of Santiago de Compostela, Santiago de Compostela, Spain; ^4^Centro de Investigación Biomédica en Red Enfermedades CardioVasculares (CIBERCV), Madrid, Spain; ^5^Cardiology Group, Health Research Institute of Santiago de Compostela, Santiago de Compostela, Spain; ^6^Heart Failure Unit, Cardiology, Hospital Universitario Central de Asturias, Oviedo, Spain; ^7^Health Research Institute of Principado de Asturias, Instituto de Investigación Sanitaria del Principado de Asturias (IISPA), Oviedo, Spain

**Keywords:** heart failure, sex, treatment, treatment-drug, adherence-compliance-persistence, ventricular assist device, heart transplantation

## Abstract

Heart disease is the leading cause of death in both men and women in developed countries. Heart failure (HF) contributes to significant morbidity and mortality and continues to remain on the rise. While advances in pharmacological therapies have improved its prognosis, there remain a number of unanswered questions regarding the impact of these therapies in women. Current HF guidelines recommend up-titration of neurohormonal blockade, to the same target doses in both men and women but several factors may impair achieving this goal in women: more adverse drug reactions, reduced adherence and even lack of evidence on the optimal drug dose. Systematic under-representation of women in cardiovascular drug trials hinders the identification of sex differences in the efficacy and safety of cardiovascular medications. Women are also under-represented in device therapy trials and are 30% less likely to receive a device in clinical practice. Despite presenting with fewer ventricular arrythmias and having an increased risk of implant complications, women show better response to resynchronization therapy, with lower mortality and HF hospitalizations. Fewer women receive advanced HF therapies. They have a better post-heart transplant survival compared to men, but an increased immunological risk needs to be acknowledged. Technological advances in mechanical circulatory support, with smaller and more hemocompatible devices, will likely increase their implantation in women. This review outlines current evidence regarding sex-related differences in prescription, adherence, adverse events, and prognostic impact of the main management strategies for HF.

## Introduction

Men and women have the same risk of developing heart failure (HF) throughout life. However, it is well-known that women develop the disease later in life. In addition, women have a higher prevalence of HF with preserved ejection fraction (HFpEF), the prevalence of which increases with age. This may partly explain the under-representation of women in pharmacologic and device therapy trials designed to treat HF with reduced EF (HFrEF) (1).

Sex based differences in pharmacokinetics and pharmacodynamics of pharmacological agents may explain the variable effects in men and women. However, given the smaller number of women included in clinical trials of HFrEF, where they represent less than one-third of the study population, we do not have accurate information. Unfortunately, the results of large clinical trials are often not analyzed separately by sex and we only have subgroup analyses so they cannot be fully extrapolated to women (2). The same under-representation applies to clinical trials for devices. Heart transplantation shows good outcomes in women, with lower long-term, cardiovascular and malignancy risk. Nevertheless, sex needs to be taken into account in order to select a suitable donor, tailor post-transplant immunosuppression and surveillance and address specific quality of live concerns and address reproductive health.

## Sex Differences in Pharmacodynamics and Pharmacokinetics

There are important sex-dependent differences in pharmacokinetics (PK) and pharmacodynamics (PD) that need to be acknowledged to understand how specific cardiovascular drugs can affect women and men differently. The differences can affect absorption, metabolism, distribution, and elimination.

### Absorption

For orally administered drugs, two main factors need to be acknowledged: compared to men, women (1) produce less gastric fluid, which can lead to a decrease in the absorption of weak acids and an increase in the absorption of weak bases and (2) have longer intestinal transit time (3, 4). The influence of estrogen on enzymes such as CYP3A can modulate intestinal transport, elimination rate, and alcohol distribution volume (3). Transdermal absorption appears to be higher in women (3).

### Distribution

Total body water is greater in men, while women have a higher proportion of adipose tissue. Therefore, distribution volume for hydrophilic or lipophilic drugs varies according to sex.

Plasmatic proteins involved in drug transport can be modulated by estrogens, resulting in a sex-dependent distribution (5, 6).

### Metabolism

Lower hepatic flow in women, sex-dependent activity of metabolic enzymes, increased proportion of adipose tissue and lower basal metabolic rate can explain differences in drug metabolism (3, 7, 8).

### Elimination

In general, glomerular filtration, tubular secretion, and tubular reabsorption are higher in men (3), however, during pregnancy, renal blood flow increases and an overall increase in glomerular filtration rate by about 50% is seen in pregnant women (9).

Liver enzyme activity decreases in presence of elevated female hormone levels which may decrease drug elimination. Therefore, metabolism can change throughout the menstrual cycle, during pregnancy, with oral contraceptives intake or after menopause (10).

## Sex Based Differences in Pharmacokinetics and Pharmacodynamics of Cardiovascular Drugs

### Digoxin

An increased risk of death in women was reported in the DIG trial. Although it may have been related to higher digoxin levels in women, it could not be proven since digoxin levels were available in less than one third of the study patients (11).

### Betablockers

Women have higher plasma levels of beta-blocker (BB) due to decreased renal clearance (Cl) and smaller distribution volume (Vd) (12). Despite this, BB have been shown to have greater therapeutic effect in men: In a chronic angina study with metoprolol, women had significantly higher heart rate and blood pressure both a rest and during exercise (13), despite similar effects on the reduction in the frequency of anginal episodes.

### Inhibitors of the Renin-Angiotensin-Aldosterone System

Sex-based differences have not been identified on the antihypertensive effects of angiotensin-converting enzyme inhibitors (ACEI), angiotensin-receptor blockers (ARB) and aliskiren (3). Although higher ARB maximum serum concentration (C_max_) and area under the curve (AUC) were found in women, the differences disappeared when adjusted for weigh (14).

### Sacubitril/Valsartan

The potential effects of age and sex on the PK of Sacubitril/Valsartan were assessed in a study that enrolled 36 subjects, 50% male and 50% female: No sex-dependent differences were found in PK (15).

### Diuretics

C_max_ and AUC of torsemide are 30–40% higher in women due to reduced elimination (16).

### Nitrates

C_max_ and AUC of isosorbide-5-mononitrate are higher in women, likely requiring weight adjustment and titration based on symptoms (17).

### Calcium-Channel Blockers

Sex-specific PK differences have been described for verapamil, nifedipine, and amlodipine. Oral clearance of verapamil and amlodipine is faster in women compared to men, due to the higher activity of CYP3A4 and lower activity of P-gp (18).

### Thrombolytics, Antithrombotics, and Anticoagulants

Warfarin dosage is strongly associated with sex, with lower requirements in women. Exogenous estrogen and testosterone can influence warfarin protein binding, so dose adjustment may be needed if hormone replacement therapy is initiated (12).

There is limited data regarding sex differences in direct oral anticoagulants (DOACs). But safety and efficacy studies suggest the importance of dose adjustment based on body weight. In a DOAC meta-analysis including 66,389 patients (37.8% women), DOACs were associated with a significantly lower risk of major bleeding in women compared to men (RR 0.86; 95% CI 0.78–0.94) and a higher risk of stroke and systemic embolism compared with men (RR 1.19; 95% CI 1.04–1.35) (19).

## Sex Representation in Heart Failure Clinical Trials

More than 30 years ago, the National Institutes of Health (NIH) established guidelines for the inclusion of women and minorities in clinical research. They recommend that clinical trials should enroll equal numbers of men and women in order to understand sex differences. Shortly thereafter, Congress approved these recommendations, and they became law. The Food and Drug Administration (FDA) published another regulation requiring detailed information by sex in clinical trials investigating new drugs, and therapies (20).

Clinical trials however, unfortunately, remain underpowered to identify statistically significant treatment effects in both sexes.

**Figure 1:**
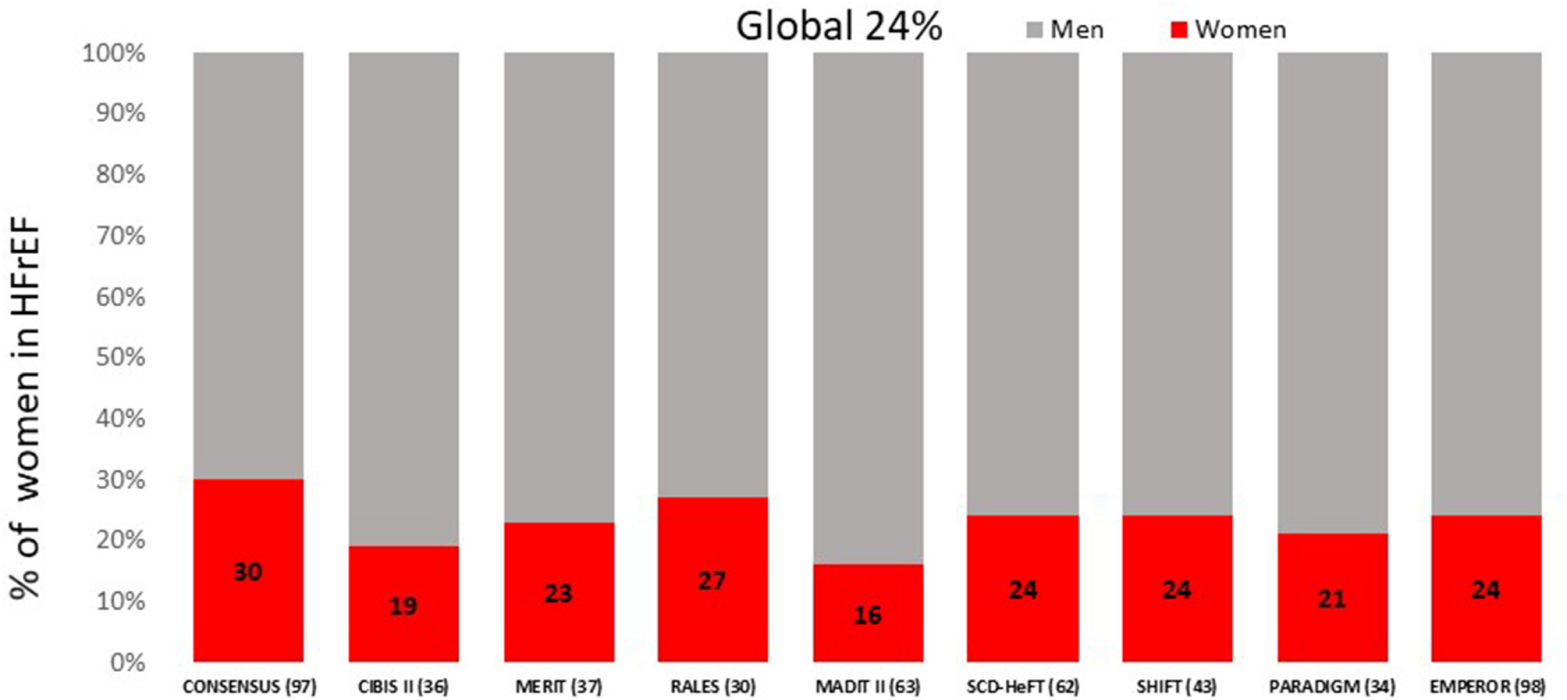
Proportion of women included in the major HFrEF clinical trials. HFrEF, Heart Failure reduced Ejection Fraction.

A recent study assessed the enrollment of women in 36 cardiovascular trials evaluating different drugs approved by FDA from 2005 to 2015. Adequacy between the percentage of women included in the trials and the prevalence of the female sex in the disease studied, was evaluated using the participation to prevalence ratio (PPR). A relationship between 0.8 and 1.2 was considered to reflect a good representation of women population. It should be noted that in the 3 HF trials included in this study women inclusion ranged from 22 to 40%. The overall PPR was 0.5, reflecting an inclusion of women in the trials well below their prevalence of the disease. More recent trials show the same pattern, ranging from 21 to 29% inclusion of women (21) (Figure 1).

**Figure 2:**
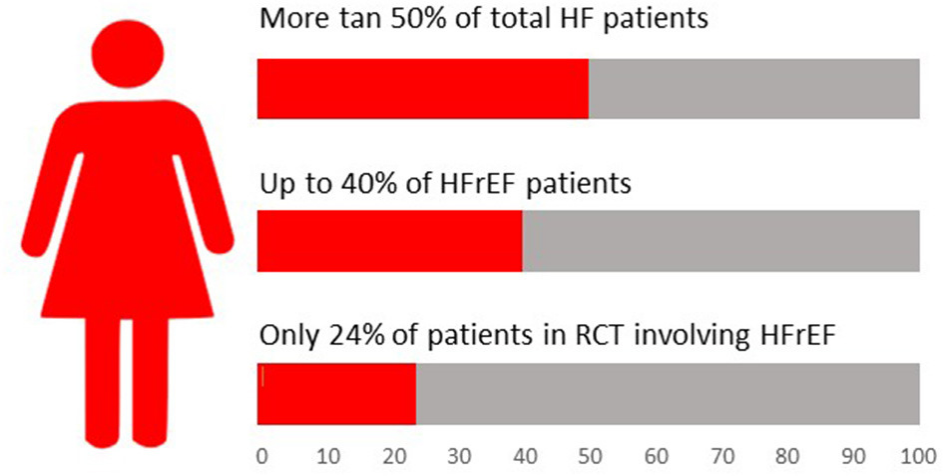
Relevance of female sex in heart failure landscape. HF, Heart Failure; HFrEF, Heart Failure reduced Ejection Fraction; RCT, Randomized Controlled Trial.

In heart failure (HF) clinical trials, women represent approximately a quarter of patients with HFrEF and over half of those with HFpEF. However, epidemiologic data demonstrate a much higher proportion of women suffering the disease in the real world (22, 23) (Figure 2).

The differences between real world proportion of women with heart failure and their representation in clinical trials may depend on a variety of factors: more comorbidities, older age in women, fulfilling exclusion criteria more frequently, lower proportion of HFrEF, less investigator counseling or less personal availability, and willingness to enroll.

### Sex Differences in Pharmacological Treatment of Heart Failure Angiotensin-Converting Enzyme Inhibitors (ACEI)

The first clinical trials with ACEI date back to the late 1980s. A sub-analysis of the SOLVD trial (24) revealed a significant reduction in the combined outcome of CHF-related death and hospitalization in men (39.5 vs. 29.7% in the placebo and enalapril arm, respectively), but not in women (38.7 vs. 37.0%). Similar findings were reported other early trials with ACEI (CONSENSUS-1, SAVE) (25). A later meta-analysis including 30 randomized clinical trials on ACEI, evaluating data from more than 5,000 men and 1,500 women, showed a significant reduction in overall mortality and HF hospitalization in men, but not in women (26). The small proportion of women included in the trials may explain the lack of positive results (27).

### Angiotensin II Receptor Blockers (ARB)

When large trials of ARB in HFrEF populations (ELITE, Val-HeFT, CHARM) explored sex-specific treatment effect they found no differences in mortality or HF hospitalization between women and men (16). ELITE II compared losartan with captopril in HFrEF patients and no difference based on sex was noted (28).

However, a population study comparing ACEI with ARB in HF, including 10,223 women (8,627 ACEI and 1,596 ARB) and 9,475 men (8,484 ACEI and 991 ARB), showed that women on ARBs had a better survival than those on ACE inhibitors, with a 31% relative risk reduction in all-cause mortality (adjusted HR 0.69, 95% CI 0.59–0.80, *p* < 0.0001). Conversely, there was no survival difference between ACEI or ARB in men (HR 1.10, 95% CI 0.95–1.30) (29).

Hormone effects on angiotensin II receptor expression or differences in adverse events may explain the potential superiority of ARB in women.

### Mineralocorticoid Receptor Antagonists (MRA)

The studies assessing the role of mineralocorticoid receptor antagonist (MRA) in HFrEF (spironolactone in RALES and eplerenone in EPHESUS), showed no sex differences in prognosis (30, 31). In subgroup analysis of the EPHESUS study, female sex was associated with a reduction in all-cause mortality, while no differences were seen in men. Nevertheless, the interaction between the sex and the treatment arm was not significant.

In the TOPCAT trial, there were no sex-specific differences in the primary outcome (32). Nevertheless, in a secondary analysis of TOPCAT, restricted to 1,767 patients (49.9% women) enrolled in the Americas, spironolactone showed a reduction on mortality, with a trend toward greater reduction in cardiovascular mortality in women compared to men (9.0 vs. 13.2%, respectively, *p* = 0.051) (33).

### Sacubitril-Valsartan

The PARADIGM-HF trial, showed superiority of sacubitril/valsartan compared to enalapril at reducing mortality and HF hospitalization in patients with HFrEF (34). In subgroup analyses, similar prognostic benefit was found for the primary endpoint in both men and women. When cardiovascular death was analyzed separately, sacubitril/valsartan showed a significant improvement in prognosis in men, but not in women (34), probably due to the small number of women included.

The PARAGON-HF trial, comparing sacubitril-valsartan and valsartan in patients with HFpEF, found no differences in primary composite end point of first and recurrent hospitalization for HF and death from CV causes. The primary composite endpoint occurred less frequently in women 0.73 (95% CI 0.59–0.90) compared to men 1.03 (0.84–1.25; *p* = 0.017) (35), primarily due to the reduction in HF hospitalization. Men were found to have a greater improvement in KCCQ-CSS than women. There were no sex differences in NYHA class, renal function, and adverse events.

In conclusion, PARAGONF_HF subanalysis suggest that sacubitril-valsartan may lead to greater reduction in HF hospitalizations in women with HFpEF.

### Betablockers (BB)

Despite the low proportion of women included in BB trials (36–38) and lack of a specific design to study sex-differences, *post-hoc* pooled analysis confirmed similar and significant benefits of BB (bisoprolol, carvedilol, metoprolol) on combined end-point of all-cause mortality and all-cause hospitalizations in both women and men (39).

Interestingly, data from the earlier US-Carvedilol-Study (40), CIBIS II trial (41) and SENIORS study (42), suggest a greater survival benefit from BBs treatment in women, but no mechanistic explanation is described.

### Ivabradine

The SHIFT trial, comparing ivabradine and placebo in patients with symptomatic chronic HFrEF (LVEF ≤35%) in sinus rhythm with heart rate >70 bpm, showed a reduction in the composite primary outcome of CV death or hospital admission for worsening HF. Subgroup analyses did not show any sex-differences in efficacy or safety of ivabradine (43).

### Sodium-Glucose Cotransporter-2 Inhibitors (SGLT2i)

In the last 6 years, several large cardiovascular outcome trials evaluated the effect of iSGLT2 in patients with type 2 diabetes and established cardiovascular disease or those with high cardiovascular risk, they have consistently shown to reduce the risk of hospitalization for heart failure (44–49).

A meta-analysis of SGLT2i including patients with type 2 diabetes enrolled in the EMPA-REG OUTCOME, CANVAS Program, DECLARE TIMI-58, and CREDENCE trials, showed (50) no sex differences in safety or efficacy outcomes (all *p* interaction ≥ 0.17).

Recently, a meta-analysis condensing two single large-scale trials (DAPA-HF trial and EMPEROR-reduced trial) in patients with HFrEF with or without diabetes assessing the effects of SGLT2i on cardiovascular outcomes have been published. SGLT2i reduced hospitalizations for HF and death, with an improvement in renal outcomes, regardless of sex and other conditions such as age, diabetes status, or baseline heart failure medications (51).

### Other Heart Failure Medications

#### Diuretics

The effects of diuretics on mortality and morbidity in chronic heart failure have not been studied in large clinical trials. There are no reported sex-related differences with diuretic therapy. Observational studies have shown a relationship between diuretics dose and mortality risk, which was maintained after adjusting for sex (52).

#### Digoxin

In the DIG trial, digoxin was associated with a significantly higher risk of death among women (adjusted HR 1.23; 95% confidence interval, 1.02–1.47), with no increased risk in men (11). Subsequent retrospective analyses showed a strong relationship between serum digoxin concentrations and survival (53). Comprehensive analysis of data indicates a beneficial effect of digoxin on morbidity (HR 0.73, 95% CI 0.58–0.93, *p* = 0.011) and no excess mortality in women at serum concentrations between 0.5 and 0.9 ng/ml, whereas serum concentrations ≥1.2 ng/ml was harmful (HR 1.33, 95% CI 1.001–1.76, *p* = 0.049).

Overall, whereas higher digoxin levels tend to increase mortality in women, low concentrations seem to be safe and associated with improved symptoms.

#### Hydralazine-Isosorbide Dinitrate

The A-HEFT trial enrolled more than 5,000 black women (41% of total cohort) with moderate to severe heart failure (NYHA class III-IV) (54) to test treatment with hydralazine-isosorbide nitrate vs. placebo.

Treatment with hydralazine and isosorbide showed a significant reduction in mortality, first heart failure hospitalization, and change in quality of life at 6 months, with no differences between men and women.

## Safety: Heart Failure Drugs and Adverse Reactions in Women

Women are known to have an increased adverse reaction (AR) to cardiovascular drugs compared to men (1.5–1.7-fold) (3) and have greater hospital admissions. Despite this fact, there is little emphasis on sex-specific differences in AR in drug trials. In a recent systematic review (55), only 7% of heart failure drug studies reported sex-based AR data. Differences in adverse events may be due to differences in absorption, body composition, drug distribution, physiological hormone changes and excretion (Table 1). These effects may be more pronounced in women with HF as they are older and have a higher prevalence of comorbidities and polypharmacy (60).

**Table 1:** Heart failure drugs pharmacodynamics, efficacy and adverse events in women compared to men.

**Drug**	**Summary**	**References**
Digoxin	- ↑ Death risk with less benefit in hospitalization. Related to higher dosage in women, considering their lower body weight.	(11, 53)
Beta-blockers	- ↑ Plasma levels with the same doses due to lower distribution volume (hydrophilic drugs) and slower clearance. - Similar or higher benefit in women.	(12, 35–39, 41, 42)
ACE-inhibitors	- Less benefit in women in clinical trials, but underrepresented (bias?). - ↑ Angioedema and cough. - Teratogenic.	(25–27, 56)
ARB	- Little evidence of more benefit in women.	(16, 28, 29, 57)
Sacubitril/valsartan	- Similar pharmacokinetic parameters. - Similar results in HFrEF hospitalizations but less reduction in CV death (underrepresented, bias?). - Less HF hospitalizations in HFpEF.	(15, 34, 35)
Mineralocorticoid receptor antagonists	- Similar or more benefit in women. - Lower withdrawal.	(30–33, 58)
Diuretics	- ↑ Serum concentration due to reduced elimination. - More electrolyte imbalance.	(16, 52, 59)
Nitrates	- ↑ Serum concentration: need to adjust for weight.	(17)
Ivabradine	- No sex differences on effectiveness.	(43)
iSGLT2	- Similar effectiveness and adverse events.	(44–51)

*ACE, angiotensin converting enzyme; ARB, angiotensin receptor blockers; HF, heart failure; HFrEF, heart failure with reduced ejection fraction; HFpEF heart failure with preserved ejection fraction; CV death, cardiovascular death; iSGLT2, sodium-glucose co-transportrer-2 (SGLT2) inhibitors; ↑, Increased*.

### Diuretics

Women experience greater electrolyte imbalance with diuretic use, which in turn increases the arrhythmic risk. For instance, women have an increased risk of drug-induced torsades de pointes (2.3-fold) related to a longer corrected QT interval induced by the effects of estradiol on potassium and calcium channel modulation (59).

### Digoxin

A post hoc analyses of the DIG study (11) showed a 20% higher death risk in women (HR 1.2, CI 1.02–1.47), with no impact on mortality in men. Moreover, digoxin showed less benefit in reducing hospitalization in women, compared to men. This may be related to dosage, since differences disappeared when dose was adjusted for ideal body weight.

### Beta-Blockers

Women present higher plasma levels of beta-blocker due to a lower distribution volume (higher percentage of fat in women, beta-blockers are hydrophilic drugs) and a slower clearance. Dosage needs to be adjusted according to these differences to prevent AR.

### Angiotensin-Converting Enzyme Inhibitors

An increased risk of angioedema and cough (2-fold) has been described in women (56). Moreover, their potential teratogenic effects need to be acknowledged in women during childbearing years.

### Angiotensin II Receptor Blockers

No significant sex-differences in risk of kidney impairment, hypotension, or hyperkalemia have been described with the use of Losartan (57).

### Mineralocorticoid Receptor Antagonists

There seems to be a higher withdrawal rate in men due to the appearance of gynecomastia (seen in 5.3% of the men) (58).

## Advanced Heart Failure Therapies in Women: Devices and Heart Transplantation

### Implantable Cardioverter Defibrillator

Implantable Cardioverter Defibrillators (ICD) have shown to reduce sudden death risk in heart failure patients with reduced ejection fraction, especially of ischemic etiology. Therefore, they have a class 1A indication according to current guidelines for primary prevention in patients with left ventricular ejection fraction (LVEF) <35% despite optimal medical therapy (61).

These recommendations are based on classical studies, such as SCD-HeFT (62), MADIT II (63), or DANISH (64), where female representation was small (23, 15, and 27%, respectively). In fact, women with heart failure (HF) are less likely to receive an ICD or counseling for ICD. In a large observational study (65) including 21,059 patients from 236 sites, 19.3% women vs. 24.6% men (*p* < 0.001) were offered ICD implantation. Of note, the same proportion of men and women underwent the implant once it was advised (63.1 vs. 62.3%, *p* = ns). In another observational study 32.2 per 1,000 men and 8.6 per 1,000 women received ICD therapy. After controlling for demographic variables and comorbidity, men were 2.44 (95% CI 2.30–2.59) times more likely to receive an ICD compared to women.

The reduced rate of ICD implantations in women may be related in part to the controversies regarding efficacy and higher risk of complications in women compared to men.

Although some device studies show a similar survival benefit after ICD implantation in both men and women, most are underpowered to study sex differences. In a metanalyses including 4,744 primary prevention ICD patients (66) (19.6% female), there was a 22% reduction in mortality in men but no benefit in women. In fact, ventricular arrythmias may be less common amongst women. The risk of sudden death was 32% lower in women compared to men in 8,337 HF patients cohort with no ICD (67). Women have consistently shown to have fewer appropriate ICD shocks. In a metanalyses (68) including 7,229 patients (22% female), women had a HR for appropriate ICD shocks of 0.63 (95% CI 0.49–0.82, *p* ≤ 0.001) compared to men and no significant benefit on mortality. In a European study (69) analyzing data from 14 registries in 11 countries (5,033 patients, 19% female), an appropriate ICD shock occurred in 8% of women vs. 14% of men, *p* = 0.0002. In the Ontario ICD Database (70) (6,021 patients, 22% female), women showed a HR 0.69 (95% CI 0.51–0.93) for ICD shock and HR 0.73 (95% CI 0.59–0.90) for appropriate antitachycardia pacing compared to men. Etiology of cardiomyopathy and scar burden may account in part for these differences. In addition, sex hormones and their influence on myocardial ion channels (Ca, K) could play a role as well (71).

ICD related complications have been reported more frequently in women. In the Ontario ICD Database (70) women were 1.9 times more likely to have a major complication within the first year after implant, including lead dislodgement. In the National Cardiovascular Data Registry (72) (38,912 initial single or dual-chamber ICD implants, 25% female) women showed higher odds of procedural complications within 90 days OR 1.30 (95% CI 1.26–1.53, *p* < 0.001). The reasons for the differences in the complication rate are unclear but could be related to delayed presentation or greater severity of illness. Smaller vessel size and a thinner walled right ventricle may explain a higher rate of pneumothorax or perforation. Increased bleeding risk have also been reported in women.

Overall, studies show sex differences in arrhythmic risk and ICD-related complications. Nevertheless, there is a risk of sudden death in women with HF and reduced LVEF that could be prevented by ICD implantation. Careful and individualized assessment is required to identify patients that would benefit the most from this therapy.

### Cardiac Resynchronization Therapy

Cardiac Resynchronization Therapy (CRT) has shown to improve functional capacity and survival amongst patients with LVEF <35%, left bundle block >130–150 ms and NYHA functional class II-IV and therefore has a class 1A indication in current HF guidelines (73). Several studies have reported under-utilization of CRT in women (74). A recent study (75) using registry data of 311,009 patients undergoing CRT implantation between 2006 and 2012 showed that only 30% were women, and women were less likely to have an ICD associated to the CRT. Interestingly however, women had a higher CRT response score compared to men. These disparities increased over the study period. In a Swedish registry (76), female sex was again associated with lower CRT implantation. Despite this, most of the studies suggest similar if not better response to CRT in women.

The CARE-HF (77) (*n* = 752, 28% female) and COMPANION (78) (*n* = 1,520, 32% female) trials demonstrated similar reduction in mortality and time to hospitalization in both sexes after CRT implant. In the MASCOT (79) trial (*n* = 393, 21% female) women showed better left ventricular remodeling and lower mortality and HF hospitalizations after adjustment for cardiovascular risk factors. Remarkably, women showed wider QRS and smaller left ventricle size at enrollment. In another study that only included patients with non-ischemic cardiomyopathy (*n* = 212, 49.5% female) CRT response among women was greater (84 vs. 58%, *p* < 0.001) than in men, despite similar baseline QRS duration (80). In fact, women showed better response compared to men at all QRS widths below 180 ms. In a retrospective analysis (81) of 619 consecutive patients (19% women) undergoing CRT implantation in a single center over a 10-year period, female sex was the only independent predictor of all—cause mortality (HR 0.44, 95% CI 0.21–0.90, *p* = 0.025) and showed a trend toward lower heart failure hospitalization. In a metanalyses (82) of 5 randomized control trials (*n* = 3,496, 23% female), QRS duration was the only independent predictor of CRT benefit. Further analysis showed the benefit was even more significant at lower height. There was a higher proportion of women amongst the wider QRS and shorter patients.

As we discussed for ICD, complication rate seems to be higher in women after CRT implant. In the MADIT-CRT trial, women were twice as likely as men to experience a major procedure-related adverse event (6.3 vs. 2.7%; *p* < 0.001) mainly related to pneumothorax, infection or bleeding. The main risk factor for complications seemed to be size and body mass index both in women and men.

Overall, women show a better response to CRT after adjusting for non-ischemic etiology of the cardiac disease. Reasons are not clearly established but this benefit could be related to a smaller ventricle size with easier conduction between the leads and presence of more typical left bundle branch block.

### Ventricular Assist Device

Mechanical circulatory support has expanded significantly in the recent years, with over 13,000 implants in the INTERMACS registry (83) between 2014 and 2018, of which only 22% were women. Technical evolution has enabled devices to become smaller and to evolve from pulsatile flow first to axial-flow and now to centrifugal flow with full magnetic levitation. This has led to a significant decrease in morbidity and mortality and increase in implantations. Left ventricular assist devices (LVAD) are now smaller and more hemocompatible. Despite this, in the MOMENTUM3 (84) trial, HeartMate3's pivotal trial, only 21% of the participants were women.

There are no sex differences in survival either in pulsatile or continuous flow devices according to INTERMACS registry (85). Complications are frequent, and include driveline infection, bleeding, pump thromboses, right ventricular failure, and neurological events. There is scarcity of data on the incidence of these complications according to sex, although several reports suggest that there might be a higher incidence of neurological events in women. In an INTERMACS registry study (*n* = 1,936, 21% female) female sex was associated with an increased risk of first neurological event (HR 1.44, 95% CI 1.05–1.96; *p* = 0.020), with no difference in other complications. In a later paper focusing on stroke rates during support with continuous-flow LVAD, female sex was also a predictor of stroke (HR 1.51, 95% CI 1.25–1.82; *p* < 0.001). The same was reported in an analysis of more than 900 HeartMate II outpatients (86) (23% female), where female sex was a risk factor for both hemorrhagic and ischemic stroke. There is lack of data on the impact of sex in stroke rate with HeartMate3 since the event rate in MOMENTUM3 was too low to derive conclusions.

There is no clear explanation as to why fewer women receive LVAD compared to men. Some aspects to consider are smaller body surface area, smaller ventricles, older age at the time of HF diagnosis and a higher prevalence of HF with preserved ejection (87), which is not suitable for LVAD support. Since there might be a risk for selection bias, we need to be aware that women benefit as much as men from this life-saving therapy, with no significant increase in complications specially with the newer generation LVAD devices.

### Heart Transplantation

Heart Transplantation (HT) is the therapy of choice to improve survival in patients with end-stage HF. Mean survival after HT nowadays is 12.5 years for the adult population and 12–21 years for the pediatric population (88). Rejection and infection are the most concerning complications in the first year post-HT, whereas the leading cause of death after the first year are coronary allograft vasculopathy and malignancy.

Women are again under-represented in the field of HT: according to the last report from the International Society for Heart and Lung Transplantation (88), only a quarter of the HT were performed in women (25% in Europe, 26% in the United States of America, and 24% in other countries). There is also a smaller proportion of women amongst the donors (37% in Europe, 30% in the United States, and 22% in other countries). Female HT receptors have shown a better life expectancy compared to male recipients: 12.2 vs. 11.4 years (*p* < 0.001) (89).

Women are younger than men at the time of listing (mean 48 year for women vs. 56 years for men), have less ischemic heart disease and more idiopathic dilated cardiomyopathy, and fewer cardiovascular risk factors such as smoking, diabetes mellitus, hypertension, or tobacco use (89). On the other hand, women are less likely to be transplanted in the higher emergency status, as they are also less frequently supported with temporary mechanical circulatory devices.

In the post HT period, women are at a lower risk of coronary allograft vasculopathy and malignancy. These differences could explain longer survival in female HT recipients.

Pre-HT sensitization and post-HT rejection risk are higher in women, related predominantly to the presence of circulating preformed HLA antibodies due to sensitization from previous pregnancies (90). Donor-recipient matching is key at the time of HT. Sex mismatch has been reported as a prognostic factor for HT outcomes, with best outcomes reported with female donor to female recipient and the worst with female donor to male recipient. A simple explanation for this fact, is the undersizing of female donor hearts when used for male recipients, however, outcome differences seem to persist even after adjustment for ventricular mass (91).

In summary, fewer women receive HT, despite their better long-term survival. Sex specificities need to be considered in the pre-HT evaluation (greater sensitization, fewer cardiovascular risk factors), at the time of transplant (sex and size donor-recipient matching) and in the long-term post-HT follow-up (increased risk of rejection but lower risk of graft vasculopathy and malignancies) (Table 2).

**Table 2:** Advanced heart failure therapies in women: summary and key messages.

**Intervention**	**Summary**	**References**
Implantable cardioverter device	- Women under represented in ICD trials (15–27%). - Less likely to receive counseling for ICD. - Fewer ventricular arrythmias and appropriate ICD shocks. - Higher rates of complications (pneumo/hemothorax).	(62–72)
Cardiac resynchronization therapy	- CRT under utilized in women (30%). - Women show better response to CRT (left ventricular remodeling, mortality and HF hospitalizations). - Wider QRS complex with classic left bundle branch block and smaller ventricles may explain better CRT response in women. - Higher rates of complications during implantation.	(73–82)
Mechanical circulatory support	- Fewer women (22%) receive MCS. - Similar overall outcomes as men in both pulsatile and continuous flow devices. - Greater risk of neurological events in women (ischemic and hemorrhagic). - New generation smaller and more hemocompatible devices could increase implant rates in women.	(83–87)
Heart transplantation	- Fewer HT are performed in women (25%). - Women have better post transplant survival, related to pre HT factors (younger age, less cardiovascular risk factors) and post HT factors (less allograft vasculopathy and malignancies). - Women have increased immunological risk (sensitization and rejection)	(88–90)

*ICD, implantable cardioverter device; CRT, cardiac resynchronization therapy; MCS, mechanical circulatory support; HT, heart transplantation*.

## Sex Differences in Adherence

Adherence to long-term therapies for chronic diseases in developed countries averages only about 50–75% (2). Inadequate adherence is associated with increased long-term mortality in patients with heart failure (92).

Few studies have aimed to assess the effect of sex on adherence to HF drugs. Granger et al. analyzed adherence among participants in the CHARM trial (*n* = 7,599) and they found that 11% were poor adherers (<80%, *n* = 836). Poor adherers were more likely to be women (12.7% of women vs. 10.2% of men; *p* = 0.002), have a higher heart rate, and a greater number of concomitant illnesses (93).

Kayiband et al. performed an inception cohort study of new users of evidence-based HF drug treatment. They included 28,067 Canadian patients (13,453 women, 14,614 men) between January 2000 and December 2008 who had a follow-up >1 year after HF drug treatment initiation. In this study women were more likely than men to be adherent to their treatment (52.8 and 50.1%, respectively, adjusted proportion ratios: 0.96, 95% CI: 0.94–0.99) (94). More recently, a retrospective, observational study was carried out in the Dutch population. Twenty-five thousand seven hundred and seventy-six patients with a diagnosis code for chronic HF between January 2012 and December 2014 were included in order to study the impact of sex differences in co-morbidities and medication adherence on a composite endpoint of all-cause mortality or HF admission. 11,259 (45%) women and 14,517 men, median age 76 and 72 years, respectively, were analyzed after a median follow-up of 3.3 years, and only slight differences in HF drugs adherence between women and men were found with no impact on the composite endpoint (95).

**Figure 3:**
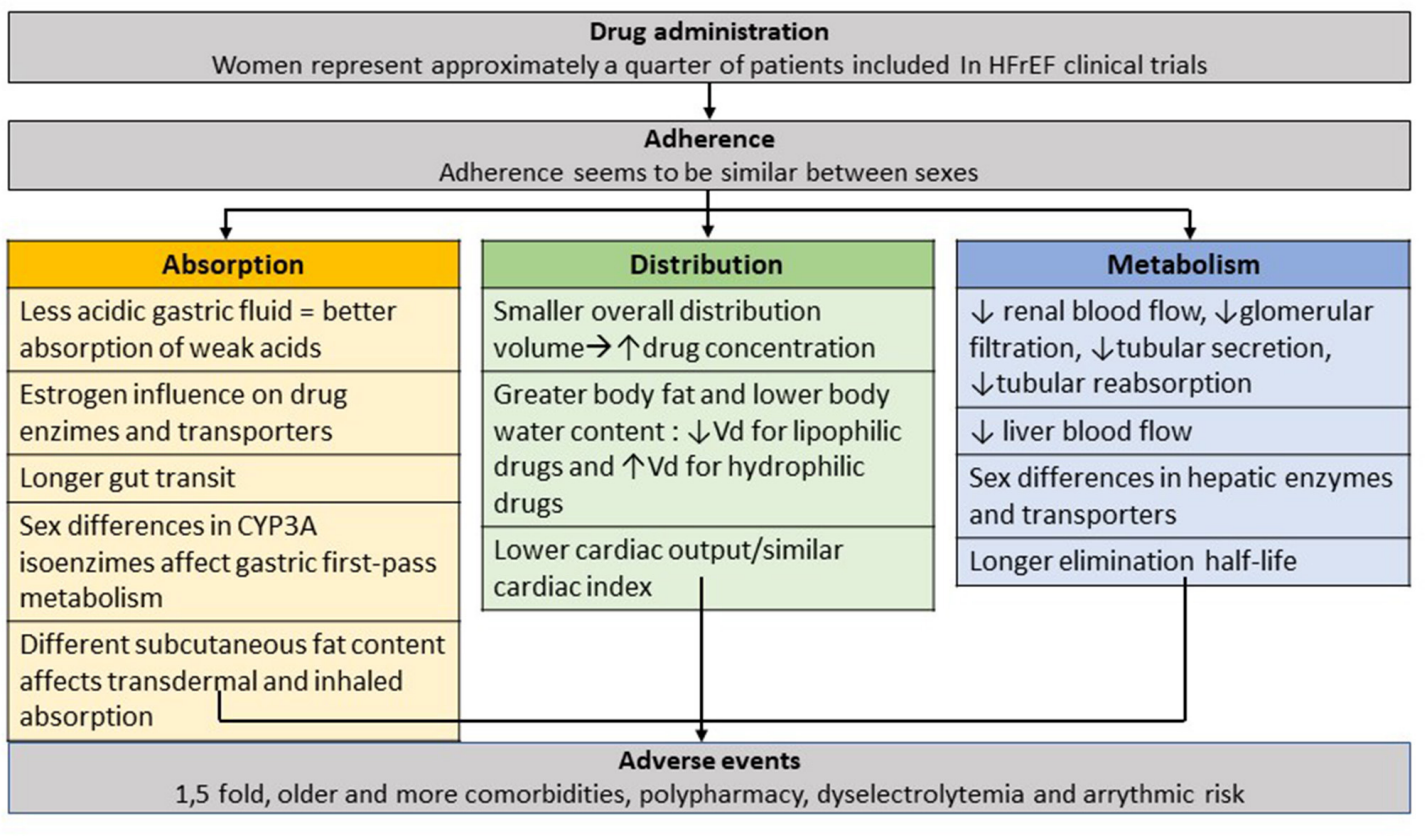
Use of heart failure drugs in women. Sex-differences on prescription, adherence, effective circulation levels and adverse events. HFrEF, Heart Failure reduced Ejection Fraction.

We can conclude that non-adherence to disease-modifying drugs is associated with an increased mortality and HF readmissions, but adherence seems to be similar between sexes (Figure 3).

## Discusion

Although women represent 50% of the world population and despite similar overall prevalence of heart failure among men and women (96) women are significantly underrepresented in clinical trials for heart failure. The trend has not changed significantly over time, with similarly low inclusion rate for women in the newer HFrEF trials. For example, women represented 30% of the study population in the enalapril CONSENSUS trial (97), in 1987, and 19% in the bisoprolol CIBIS II trial in 1999 (36) and represented 24% of the study population in the EMPEROR trial (98), published in 2020. This may be due to a higher proportion of women with HFpEF, older age and more comorbidities, limiting their chances of being included in HFrEF trials. In HFpEF trials, such as PARAGON (99) or TOPCAT (32), women represent 50% of the study population, which is higher compared to HFrEF trials, but still low in comparison to the percentage of women in the population with HFpEF. In fact, HF is the discipline of cardiology in which women are most underrepresented (21) in clinical trials.

Women are generally more symptomatic than men when they present with HFrEF (100), which could in turn be related to a later medical contact, minimization of symptoms, acceptance of a poorer quality of life (101) and a prioritization of their social role as caregivers. Due to underrepresentation in clinical trials, we have limited information on the efficacy and adverse effects of therapies in women. In a recent study on the use of guideline recommended therapy for HF and its titration, women had a similar proportion of HF drug and dose prescription compared to men, at baseline and at 1-year follow up. Considering the differences in adherence, absorption, metabolism, body weight and adverse events between men and women, it would be reasonable to establish a more tailored therapy according to sex. The main limitation for an individualized approach remains to be the lack of reliable data. Santema et al. (102) showed how, despite achievement of similar target doses of HF guideline recommended therapy in men and women, the lowest hazards of death for men occurred at 100% of the recommended dose, whereas women showed 30% lower risk at 50% of the recommended dose, with no further decrease in risk at higher doses.

Women are also less likely to receive lifesaving therapies such as LVAD (103) and HT. LVAD therapy has shown similar survival benefits in women compared to men, but women tend to be more unstable at the time of implant, with worse INTERMACS profiles and more severe tricuspid regurgitation (104). In the HT arena, despite similar overall survival, women are more likely to receive hearts from higher risk donors (105).

Reproductive health counseling, teratogenic effect of HF medications and pregnancy management for women with HF are some important topics that affect women uniquely and that need to be a focus for future research and discussion, especially for those in need of advanced HF therapies and devices.

## Conclusions and Future Perspectives

Despite high prevalence of HF in women, there is lack of data on the use of drugs and HF therapies, with limited enrolment in randomized control trials and limited access to lifesaving strategies. Future trials should focus on greater enrollment of women in heart failure therapeutics and devote resources to understand the pathophysiology of the sex differences and disparities in access to advanced therapies.

## Author Contributions

All authors listed have made a substantial, direct and intellectual contribution to the work, and approved it for publication.

## Conflict of Interest

The authors declare that the research was conducted in the absence of any commercial or financial relationships that could be construed as a potential conflict of interest.

## Glossary

AAG: alpha-1 acid glyconprotein
ACEI: angiotensin-converting enzyme (ACE) inhibitors
AR: Adverse reaction
ARB: angiotensin-receptor blockers
AUC: area under the curve
BB: Betablockers
BMR: basal metabolic rates
BSA: body surface area
CI: Confidence Interval
CI: cardiac index
Cl: clearance
CO: cardiac output
CRT: Cardiac Resynchronization Therapy
CYP3A4: cytochrome P450 3A4
CV: cardiovascular
DOACs: direct oral anticoagulants
FDA: Food and Drug Administration
HF: heart failure
HFpEF: Heart Failure preserved Ejection Fraction
HFrEF: Heart Failure reduced Ejection Fraction
HR: Hazard Ratio
HT: Heart transplantation
ICD: Implantable Cardioverter Defibrillator
LVEF: left ventricular ejection Fraction
LVAD: Left Ventricular Assist Device
MRA: Mineralocorticoid receptor antagonists
NIH: National Institutes of Health
PK: pharmacokinetics
PD: pharmacodynamics
PPR: participation to prevalence ratio
SGLT2i: Sodium-glucose cotransporter-2 inhibitors
Vd: volume distribution
